# Data linkage in public health research: a scoping review of ethical, legal and societal aspects surrounding administrative data

**DOI:** 10.23889/ijpds.v9i5.2716

**Published:** 2024-09-18

**Authors:** Félix Jesus Neves, Mauricio Lima Barreto, Valerie Wells, Martha Silveira, Bethânia Araújo Almeida

**Affiliations:** 1 Federal University of West of Bahia; 2 Centre for Data and Knowledge Integration for Health, Oswaldo Cruz Foundation; 3 Medical Research Council; 4 Chief Scientist Office, Social and Public Health Sciences Unit, School of Health and Wellbeing, University of Glasgow; 5 Gonçalo Moniz Institute, Oswaldo Cruz Foundation

## Introduction

The application of data linkage for scientific research purposes in the public health sphere requires using personal and sensitive data on an individual level while results are analyzed from a population perspective. Considering this, ethical, legal and social issues (ELSI) must be considered. In this sense, our study aims to better understand how ELSI surrounding administrative data linkage use in public health has been addressed in the scientific literature.

## Methodology

Our scoping review is based on the Joann Briggs Institute Manual for Evidence Synthesis and will be reported according to the Preferred Report Items for Systematic Reviews and Meta-Analyses – Scoping Review Guideline. Two independent authors searched for articles published between January 2013 and December 2023 in the PubMed/MEDLINE, Web of Science, EMBASE and LILACS databases, as well as in Scielo and Google Scholar platforms. Keywords used were related to ‘data linkage’, ‘administrative data’, ‘ELSI’, “public policies’ and ‘public health’ terms. Articles were screened initially by title and abstract, then by full-text read.

## Results

After duplicate removal and articles screening, two independent authors are performing data extraction considering ethical, legal and regulatory basis as well as procedures for data access; data management process including record linkage; type of administrative data linked; data protection strategies; and data subjects rights.

## Conclusions

This scoping is underway, we intend to have our final results by April to present at the conference, in September.

